# Nitrogen isotope composition of amino acids reveals trophic partitioning in two sympatric amphipods

**DOI:** 10.1002/ece3.6734

**Published:** 2020-09-23

**Authors:** Matias Ledesma, Elena Gorokhova, Henry Holmstrand, Andrius Garbaras, Agnes M. L. Karlson

**Affiliations:** ^1^ Department of Ecology Environment and Plant Science (DEEP) Stockholm University Stockholm Sweden; ^2^ Department of Environmental Science and Analytical Chemistry (ACES) Stockholm University Stockholm Sweden; ^3^ Mass Spectrometry Laboratory Centre for Physical Science and Technology Lithuania; ^4^ Stockholm University Baltic Sea Centre Stockholm Sweden

**Keywords:** amino acids, Baltic Sea, compound‐specific stable nitrogen isotope analyses, reproductive status, resynthesis index, trophic level

## Abstract

According to ecological theory, two species cannot occupy the same niche. Using nitrogen isotope analyses (δ^15^N) of amino acids, we tested the extent to which two sympatric deposit‐feeding amphipods, *Monoporeia affinis* and *Pontoporeia femorata*, partition their trophic resources. We found that trophic position (TP) and resynthesis index (∑V; a proxy for degradation status of ingested material prior to assimilation by the consumer) differ between species. The surface‐feeding *M. affinis* had higher TP and intermediate ∑V, both pointing to a large contribution of metazoans in its diet. *P. femorata*, which feeds in the subsurface layers, had lower TP and a bimodal distribution of the ∑V values, supporting previous experimental evidence of a larger feeding niche. We also evaluated whether TP and ∑V values have consequences for amphipod fecundity and embryo viability and found that embryo viability in *M. affinis* was negatively linked to TP. Our results indicate that the amino acid‐δ^15^N data paired with information about reproductive status are useful for detecting differences in the trophic ecology of sympatric amphipods.

## Graphical abstract

This is a field study on niche partitioning in sympatric amphipods using the amino acid nitrogen‐isotope method. Fecundity and embryo viability were measured for each individual analyzed for isotopes, meaning that ecological and physiological causes behind observed patterns could be tested and discussed in a unique way. We demonstrate that the amino acid method was useful in detecting differences in trophic ecology between two deposit‐feeding species, but that it is crucial to include measurements of physiological status when interpreting nitrogen isotope data in amino acids of consumers.

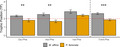

## 1 INTRODUCTION

According to what is often referred to as the principle of competitive exclusion, no two species can have the same niche; the less effective competitor will be extinct from an area (Gause, 1932; Grinnell, 1904; Volterra, 1928). The basis for coexistence is, thus, the niche differentiation which is often achieved through resource partitioning (e.g., the theory of limiting similarity, Abrams 1983).

In the species‐poor Baltic Sea, the two co‐existing and morphologically similar deposit‐feeding amphipods, *Monoporeia affinis*, a glacial relict, and *Pontoporeia femorata*, of marine origin, dominate in abundance the benthic communities at soft sediment bottoms. Like all deposit‐feeders in this area, the spring phytoplankton bloom constitutes the largest annual food input (Cederwall 1977; Elmgren, 1978; Lehtonen & Andersin, 1998). Experimental studies have demonstrated that *M. affinis* and *P. femorata* display both habitat and resource partitioning, with *P. femorata* burying deeper down in the sediment (Hill & Elmgren, 1987) and feeding more on aged organic matter when *M. affinis* is present (Byrén, Ejdung, & Elmgren, 2006). However, when released from the competition with *M. affinis*, *P. femorata* also feeds on the fresh material (Karlson, Nascimento, Näslund, & Elmgren, 2010), indicating an asymmetrical competition between the two species and a broader trophic niche of the latter. The trophic ecology in both species is well studied during the productive seasons (spring: van de Bund, Ólafsson, Modig, & Elmgren, 2001; Byrén et al., 2006; summer: Karlson, Nascimento, & Elmgren, 2008 ; Karlson et al., 2010; Karlson, Gorokhova, & Elmgren, 2014); however, less is known about resource partitioning during low productive season (winter).

The trophic position (TP) of consumers is an important component of the trophic niche; however, TP is not easily quantified in omnivorous species, such as benthic deposit‐feeders, because it is difficult to accurately sample the food items which are assimilated. The nitrogen isotope composition (δ^15^N) of a consumer is indicative of its trophic position, since the heavy isotope is enriched for every trophic transfer (Minagawa & Wada, 1984). The so‐called baseline δ^15^N (the ultimate nitrogen source) can, however, vary considerably between ecosystems and regions (Hansson et al., 1997), confounding the TP estimate, if not adjusted for this variability (Post, 2002). Field studies carried out before and after a bloom of nitrogen‐fixing cyanobacteria that have a uniquely low δ^15^N signal showed that *M. affinis* but not *P. femorata* assimilates this resource, implying that *P. femorata* is primarily a subsurface feeder not using this freshly settled summer bloom material (Karlson et al., 2014). However, differences in growth rates can also affect isotope composition (Gorokhova 2018), which could mask shifts in the diet sources. The fact that *M. affinis* is more fecund and has a more variable metabolic rate and lipid content during the year compared to *P. femorata* (Cederwall, 1979; Hill, Quigley, Cavaletto, & Gordon, 1992) could hence affect variability of its isotope composition.

As a solution to the problem with multiple potential food sources for omnivores and variability in the isotope baseline, analysis of δ^15^N in specific amino acids (in contrast to the traditional bulk measurements of an entire organism or a certain tissue) has become increasingly popular for TP assessment and for encoding the baseline signal (Chikaraishi et al., 2009). The theory behind the use of nitrogen in trophic ecology is based on the physical and chemical characteristics of amino acids. With each trophic transfer, some amino acids (AAs), so‐called trophic AAs (e.g., alanine, valine, and glutamic acid), change their δ^15^N value as a result of chemical reactions involved in assimilation and transformation of AAs, that is, metabolic fractionation involving amination and de‐amination reactions (Chikaraishi et al., 2009). The other group of AAs is source AAs (e.g., phenylalanine, hereafter Phe) that change only little with trophic transfer (McClelland & Montoya, 2002). Therefore, the relationship between the trophic AAs and source AAs in a consumer provides information on the trophic position and the ultimate nitrogen source (baseline) of the diet (Chikaraishi et al., 2009). In theory, this should yield a more accurate TP estimate compared to the bulk method.

Isotope composition of AAs in organisms is however known to be influenced by the mode of nitrogen excretion in the consumer and by diet quality (e.g., a mismatch in AA content between diet and consumers increases de‐amination in trophic AAs; Lorrain et al., 2009; McMahon, Thorrold, Elsdon, & McCarthy, 2015; McMahon & McCarthy, 2016; Nielsen, Popp, & Winder, 2015). Also, toxic exposure, leading to compromised physiological status, has been linked to enriched bulk δ^15^N values in crustaceans including *M. affinis* (Ek, Karlson, Hansson, Garbaras, & Gorokhova, 2015; Karlson, Reutgard, Garbaras, & Gorokhova, 2018), and one would expect that these effects on the δ^15^N values in trophic AAs would be even more pronounced, because of their greater metabolic fractionation. However, few studies include information on physiological status of consumers when interpreting TP estimates based on AA‐δ^15^N values (but see, e.g., Ek et al., 2018; Lübcker, Whiteman, Millar, de Bruyn, & Newsome, 2020; McMahon & McCarthy, 2016).

In addition to the TP analysis, δ^15^N variability among the trophic AAs, the so‐called resynthesis index (∑V) (McCarthy, Benner, Lee, & Fogel, 2007), can be used as a measure of relative degradation of organic matter (OM) by different groups of consumers (i.e., heterotrophic resynthesis/reworking of material). This index has recently been used by Kędra, Cooper, Zhang, Biasatti, and Grebmeier (2019) to interpret the trophic ecology in sediment‐living macrofauna. Sediment is a complex matrix, where microbial metabolism plays a fundamental role in OM biogeochemistry, ^15^N fractionation, and the resulting AAs‐δ^15^N values. The nutritional quality of the OM may decrease during degradation (Dauwe, Middelburg, Herman, & Heip, 1999), although preconditioning of OM by various protists may also improve food quality for multicellular consumers (Karlson et al., 2014; Tenore, Tietjen, & Lee, 1977), such as amphipods. The rationale behind the ∑V index calculations is that heterotrophic AA resynthesis by microbes preferentially cleaves carbon‐^14^N bonds in selected AAs, thus producing a more variable δ^15^N AA pattern. As a result, higher ∑V values are associated to microbial resynthesis (McCarthy et al., 2007), while nondegraded autotrophic material will have very little variation in the δ^15^N AA pattern. The intermediate ∑V values resulting from metazoans resynthesis represent not only autotrophic source signatures, but also subsequent alteration due to trophic transfer where biosynthetic pathways are relatively simple (Hare Fogel, Stafford, Mitchell, & Hoering, 1991; McCarthy, Benner, Lee, Hedges, & Fogel, 2004; McClelland & Montoya, 2002).

As a complement to TP and ∑V calculations, the AA threonine (Thr) has been suggested as a proxy for trophic transfer (Styring, Sealy, & Evershed, 2010). Low Thr δ^15^N values with increasing TP are a pattern found in both vertebrates and invertebrates (Bradley et al., 2015; McMahon 2015; Mompeán, Bode, Gier, & McCarthy, 2016). Finally, the source AA phenylalanine (Phe) might varying δ^15^N across the sediment depth with varying OM quality. Recently, Kędra et al. (2019) demonstrated a positive association between Phe‐δ^15^N for subsurface feeders and the heterotrophic degradation status of the OM in sediments similar to our system.

The aim of this study was to evaluate trophic differences between *M. affinis* and *P. femorata* using their TP and ∑V values obtained by the AAs‐δ^15^N approach. Based on the earlier experimental evidence for the resource partitioning in *M. affinis* and *P. femorata* feeding on fresh and aged OM, respectively, we expected these amphipods to differ in TP, Thr‐δ^15^N, Phe‐δ^15^N, and ∑V. The top few centimeters of the sediment is inhabited by diverse meiofauna communities, including juvenile copepods and nematodes (Nascimento, Karlson, & Elmgren, 2008; Ólafsson & Elmgren, 1991) and have higher bacterial abundance (Llobet‐Brossa, Rosselló‐Mora, & Amann, 1998) compared to the refractory organic matter in the deeper sediment layers. Therefore, we predicted that compared to the sub‐surface‐feeding *P. femorata*, *M. affinis* that feeds in the upper part of the sediment would have (a) more depleted δ^15^N values in Phe (sensu Kędra et al., 2019); (b) higher TP and lower Thr‐δ^15^N values indicating a more omnivorous diet due to consumption of, that is, a considerable amount of metazoans; and (c) intermediate ∑V values (McCarthy et al., 2007) reflecting such omnivorous feeding. Moreover, we expected these differences to depend on the OM content in the sediment and expected that higher OM in the sediment would be associated with the higher bacterial activity and the higher Phe‐δ^15^N in the sediment (Kędra et al., 2019). Finally, to explore potential outcomes of expected differences in diet for reproductive success, we related TP, Thr‐δ^15^N, and ∑V values to the reproductive status of the amphipods measured as fecundity and embryo viability. Both species are used in the national monitoring program as sentinel species of environmental contaminants in sediments because the embryo development during winter is sensitive to toxic exposure and can be rather easily assessed when embryos are still in the females brood pouch (Helcom, 2018; Sundelin & Eriksson, 1998). This monitoring sampling therefore providing a unique opportunity to link individual data on reproductive status with information on diet in this study. We explored potential relationships both within species and for the amphipod community consisting of both populations because *P. femorata* has been shown to have a similar feeding behavior to *M. affinis* when the latter is absent (Byrén et al., 2006; Hill & Elmgren, 1987; Karlson et al., 2010).

## 2 METHODS

### 2.1 Amphipod field sampling

The upper 2–3 cm sediment and the amphipods living there were sampled with a bottom sled (Blomqvist & Lundgren, 1996) during the yearly survey within the Swedish National Marine Monitoring Program (SNMMP) for monitoring biological effects of contaminated sediments. The sampling was conducted on 17 January 2017 in the Askö Island region, southern Stockholm archipelago (Figure S1), which is a reference region in SNMMP. Amphipods from stations 6020 (36 m; 58°48′39,96″N, 17°36′35,28″E), 6025 (38 m; 58°47′27,96″N, 17°43′51,96″E), and 6,022 (46 m; 58°44′40,56″N, 17°48′45,00″E) were collected by careful sieving of the sediment (mesh size 1 mm). These stations differed in the organic content of the sediment (Figure S2). The amphipods were immediately placed in jars filled with ambient sea water and transported to the laboratory, where they were kept at in situ temperature (4°C), in darkness with a regular water replacement to prevent hypoxia. Gravid females of *M. affinis* and *P. femorata* were used for the analyses; no *M. affinis* was available at station 6022. Total organic carbon (TOC) content of 20 mg aliquots of oven dried (60 C°) sediment was analyzed in a Leco‐CHN analyzer (with EDTA as standard) at the accredited chemical laboratory at Department of Ecology, Environment, and Plant Sciences, Stockholm University. Acidification of Baltic sediments is not necessary as <0.1% of the carbon is inorganic (Walve, J, Stockholm University, pers. comm).

### 2.2 Reproductive variables, sample classification, and preparation for chemical analyses

To determine fecundity and embryo viability, 81 gravid females were analyzed according to (Sundelin & Eriksson, 1998). Briefly, the number of embryos in the brood and number of any aberrant embryos as well as the aberration type were recorded and expressed as a percentage of the total number of embryos in the brood. In addition, the presence of parasites in the females was noted. The de‐brooded females (*M. affinis:* *n* = 39 and *P. femorata:* *n* = 42) were freeze‐dried, weighed to determine the individual dry body mass (BM; mg), and used for AA extraction and stable isotope analysis (hereafter AA‐δ^15^N). Due to the small BM (mean ± *SD*; 1.44 ± 0.28 mg for *M. affinis*; and 2.35 ± 0.80 for *P. femorata*), females of similar reproductive status were grouped to obtain sufficient biomass for the AA‐δ^15^N analysis; the target sample mass was 5 mg of dry body mass per sample. In this grouping, we considered species, station, fecundity, and percentage of the aberrant embryos. After the grouping, each sample contained between 2 and 8 females, with more individuals per sample for the smaller *M. affinis*. This procedure resulted in 8 (coded as M1‐M8) and 11 (coded P1‐P11) samples for AA‐δ^15^N analysis in *M. affinis* and *P. femorata*, respectively (see Appendix S1, Figure S3).

### 2.3 Amino acid extraction

The samples were homogenized to a fine powder and hydrolyzed together with an internal standard, norleucine (Nle), in glass vials using 6 M hydrochloric acid (HCI) for 70 min at 150°C. Thereafter, the samples were evaporated until dryness under a gentle stream of N_2_ at ~80°C, re‐suspended in 0.01 M HCI, and loaded on cation exchange columns (Dowex 50WX8, Bio‐Rad Laboratories) for purification. To elute the amino acids fraction from columns, a 10% NH_3_ solution was used_,_ and the mobile phase was evaporated until dryness and stored at −20°C. To volatilize free AAs, the derivatization step was applied according to Yarnes and Herszage (2017), with slight modifications. The procedure consisted of adding 100 µl 0.4 M HCl to dissolve the powdered sample, followed by addition of 35 µl methanol and 30 µl pyridine. Then, 15 µl of methyl chloroformate was added to initiate the reaction. Thereafter, 100 µl of chloroform was added, and the sample was centrifuged. The organic phase was transferred to a gas chromatography (GC) vial with a 250‐µl insert.

### 2.4 Stable isotope analyses

The δ^15^N values in the individual AAs of a single 2‐µl injection were measured on a Thermo gas chromatography/ combustion/ isotope ratio mass spectrometry (GC‐C‐IRMS) system consisting of a trace GC chromatograph, IsoLink IV combustion interface with a nickel/ copper oxide reactor, a Conflo IV unit for introduction to the Delta V Plus mass spectrometer, and a PTV injector. A SGE Analytical Science, ID: BPX70 capillary column (30 m × 0.25 mm), was used for the chromatographic separation. Standard operating conditions were applied with regard to reactor temperature (1,030°C), and the evolved CO_2_ was cryogenically removed to avoid isobaric interference by CO^+^ ions on mass 28. For peak integration, we used individual background type since the chromatogram peaks were within the optimal intensity range (see Appendix S1 for details on quality control of data); for the actual peak measurement, the seed oxidation method was used. Data were extracted and analyzed with the ISODAT software packages (3.0). All analyses were performed at the Department of Environmental Science and Analytical Chemistry (ACES), Stockholm University.

### 2.5 Data analysis

The chromatograms were visually inspected before extracting the isotope values. Acceptable peaks in all samples except two for *P. femorata* samples were found for the following trophic AAs: alanine (Ala), valine (Val), and pyro‐glutamic acid (Glu) and for the source AA, phenylalanine (Phe) resulting in 17 samples. For eight of the samples, acceptable peaks were also found for the following trophic AAs: proline (Pro), leucine (Leu), isoleucine (Ile), and threonine (Thr), the latter which has its own category often referred to as “metabolic” AA in vertebrates (Germain,  2013; McMahon et al., 2015; O’Connell, 2017). Correction factors based on regression analysis of standards were applied when necessary (see Appendix S1 for details; Table S1, Figure S4). Due to the utilization of methyl chloroform in the derivatization method in combination with low pH, converted pyro‐glutamic acid is the primary product of glutamic acid in the derivatized sample, instead of a mixture containing approximately equal quantities of both products. Therefore, pyro‐glutamic acid reflected the underivatized partner signature of the glutamic acid more accurately and was used instead of glutamic acid for the TP calculation.

Trophic position (TP) was calculated using two alternative approaches: single pair of AAs (e.g., ^15^N_Glu_ and ^15^N_Phe_) following the method of Chikaraishi et al. (2009) (Equation 1); and average values of multiple trophic and source AAs, using the method proposed by Nielsen et al., (2015) (Equation 2):

(1) $$\text{TP}\frac{x}{y}=\frac{\left(\delta^{15}N_{x}‐\delta^{15}N_{y}‐\beta_{x}{/}_{y}\right)}{\left(\Delta_{x}‐\Delta_{y}\right)}+1$$

(2) $$\text{TP}\frac{x}{y}=\frac{\sum \left(\delta^{15}N_{\mathrm{xi}}\pm \text{SD}\delta^{15}N_{\mathrm{yxi}}\right)/X‐\sum \left(\delta^{15}N_{\mathrm{yj}}\pm \text{SD}\delta^{15}N_{\mathrm{yj}}\right)/Y‐\beta_{x/y}\pm \text{SD}\beta_{x/y}}{\left(\Delta_{x}‐\Delta_{y}\pm \text{SD}\Delta_{x}‐\Delta_{y}\right)}+1$$

where N*_xi_* is the δ^15^N values of trophic AA*_i_*, and *N_yj_* is the δ^15^N values of source AA*_j_*. The letters in subscript *_i_* and *_j_* corresponds to the different trophic and source AAs respectively, in the equation. *β_x/y_* corresponds to the difference between the δ^15^N values of trophic AAs (*x*) and source AAs (*y*) in primary producers, and Δ_x_ and Δ_y_ are the ^15^N trophic enrichment factors (TEF) for each AA(s)*_x_* and *_y_*, respectively. Values for *β_x/y_* and TEF differ between the equations; in Equation 1, TP was calculated from each pair of AAs, Glu‐Phe, Ala‐Phe, and Val‐Phe using the following values for β_x/y_ and TEF (3.4 and 7.6), (3.2 and 5.7), and (4.6 and 4.6). In Equation 2, the values were 2.9 for β_x/y_ and 5.9 for TEF.

Using multiple AAs in the TP calculations (Equation 2) has been suggested as more accurate (Bradley et al., 2015; Décima, Landry, Bradley, & Fogel, 2017; Nielsen et al., 2015) as long as the analytical precision of each AA used for the multiple TP calculation is acceptable. We used Glu and Ala as trophic AAs and Phe as the source AA. We did not include Val in TP calculations since it has been shown to have poor predictability of ^15^N fractionation (Bradley et al., 2015; Downs, Popp, & Holl, 2014; Hannides, Popp, Landry, & Graham, 2009).

To assess the remineralization status of the food sources in each species, the ∑V index, a proxy for heterotrophic bacterial or metazoan resynthesis, was calculated according to McCarthy et al. (2007) using three AAs (Equation 3):

(3) $$\sum V=\sum \left|AA_{i}‐Avgtrp\right|/n$$

where ∑V is the absolute value of the mean deviation in δ^15^N of each trophic AA*_i_* over the grand mean of all trophic AAs *(Avg trp*) divided by *n* (total number of trophic AAs used in the calculation). As trophic AAs, we used: Glu, Ala, and Val (for those eight samples mentioned before where acceptable peaks were obtained for other AAs, we compared ∑V calculated based on 3 versus 6 AAs and found no difference (Table S2)). The defined ranges of ∑V values in phytoplankton and metazoans are 0–1 and 1–2, respectively, whereas ∑V values >2 indicate substantial bacterial resynthesis (McCarthy et al., 2007).

### 2.6 Statistics

Species‐specific difference in the δ^15^N of the source AA Phe and in the AA Thr, the TP values, and the ∑V values were evaluated using different methods. Phe‐δ^15^N, Thr‐δ^15^N, and TP values were compared between the species using unpaired *t* tests. The comparison of Thr‐δ^15^N between the species was conducted with a lower sample size (*n* = 4 for each species since peaks were not always acceptable in each sample). The ∑V values showed an approximated bimodal distribution for *P. femorata* and, therefore, we used Hartigan's dip‐statistics (HDS; Hartigan & Hartigan, 1985) to measure departure from the unimodality. Differences in subgroups of *P. femorata* (based on HDS) were thereafter tested for differences in fecundity, TP and %VE using *t* tests and chi‐square test, respectively. To test for a station effect on the Phe‐δ^15^N values in amphipods and the organic carbon content in sediment, we used one‐way ANOVA followed by Tukey post hoc test. In addition, one‐way ANOVA and Kruskal–Wallis were used to evaluate station effect on the δ^15^N values for each of the three trophic AAs (Ala, Val, and Glu). Shapiro–Wilks test and *F* test were used to test for normality of the distribution and homogeneity of variance when the variables tested had 2 levels. Variables with more than two levels were visually inspected and homogeneity of variance tested using Levene's test. Tests on isotope and reproductive data were conducted both separately for each species (*M. affinis*, *n* = 8, *P. femorata* = 9) as described above but also tested on pooled data of amphipods (referred to as *amphipods community*; *n* = 17) as described in the introduction (i.e., the two species can be considered redundant in their trophic ecology depending on their densities).

To evaluate the relationships between the reproductive status and TP, standardized major axis (SMA) regressions (Warton, Wright, Falster, & Westoby, 2006) were performed for species‐specific and amphipods community data. SMA was used because both variables, reproductive status, and TP have associated errors; moreover, the TP estimate can be confounded by physiological status of the consumer (Gorokhova 2018; Ek et al., 2015; Karlson et al., 2018). Spearman rank correlation test was used to obtain the p‐value for the relationship. Thr‐δ^15^N values against the reproductive status, TP and ∑V, were analyzed with a Spearman rank correlation. The amphipods community ∑V against the reproductive variables and against TP were analyzed with generalized additive models (GAM; Hastie & Tibshirani, 1990). The reason for using GAMs here was that the pattern was clearly nonlinear and that higher fecundity and TP with intermediate ∑V values could be expected since the latter indicates reworked phytodetritus by metazoans that are more abundant in surface sediments and that in turn may contain the highest nutritional values during the winter season when there is no freshly deposited phytoplankton material available. Community consisting of the two populations is modeled as one since we assume that the main factor affecting the feeding behavior is the competition and not the physiology (see Karasov & Diamond, 1988). Gamma family was applied to data on fecundity and TP, with log and identity link function, respectively. Smoothing parameter was estimated using generalized cross‐validation and included in the model to estimate the nonparametric function.

In the larger dataset (prior to grouping females of similar reproductive status for isotope analyses as described under sample classification), differences in the reproductive variables (fecundity and embryo viability, %VE) and biomass (BM) between the species and the species‐specific relationships of reproductive variables to BM as well as influence of station effect were evaluated. The distribution of all variables were visually explored, and when necessary, HDS was used to test whether the distribution deviated from unimodality. Species effect on fecundity and BM was tested using Mann–Whitney U test, while %VE was tested using Fisher's exact test. Relationships between BM and fecundity as well as %VE for each species were evaluated using SMA regression as described above. Since there were differences in the organic matter between the stations (Figure S2), we used station as a categorical factor in the regression analysis when a significant correlation was observed between the variables.

When assumptions on normality or homoscedasticity for SMA regression were not met, the data were transformed. Percentage viable embryos were always Box‐Cox transformed, both for species‐specific and amphipods community data; fecundity and TP values were log‐transformed when the relationship between them was evaluated. For individual data, BM was Box‐Cox transformed when the BM‐%VE regression was evaluated. All data were analyzed using the R software environment 3.4.3 ( R Core Team, 2020), and the following R packages: smatr (Warton, Duursma, Falster, & Taskinen, 2012), mgcv (Wood, 2019), diptest (Maechler & Ringach, 2016), and MASS (Ripley et al., 2019).

## 3 RESULTS

### 3.1 Species and station effects on isotope data and derived metrics

There was no significant difference in Phe‐δ^15^N between the species (*M. affinis*: 5.59 ± 1.33 and *P. femorata*: 6.16 ± 1.00; unpaired *t* test, *t*_1,15_ = −1.012, *p* > .3). Phe‐δ^15^N values (pooled for both species) differed, however, significantly among the stations (F_2, 14_ = 3.778, *p* < .05; Figure S5), with significantly lower values for stn 6025 compared to stn 6020 (*p* < .05). No significant differences between the stations were found for the trophic AAs (Table S3). A significant variation in the sediment total organic carbon content among the three stations was found (one‐way ANOVA, *F*_2, 20_ = 30.210, *p* < .001; Figure S2); however, stn 6,025, which had the lowest Phe‐δ^15^N, had intermediate levels of the organic carbon content (Figure S2).

**Figure 1:**
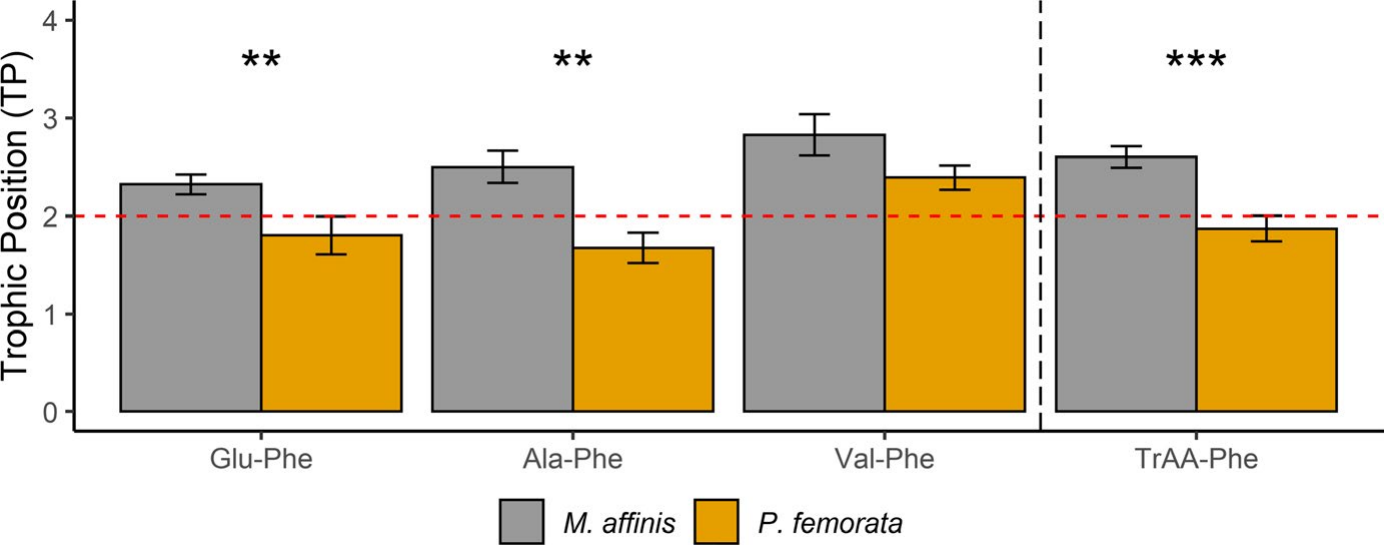
Unpaired *t* tests were used to test for difference in the TP (trophic position; mean and standard error) between *M. affinis* (*n* = 8) and *P. femorata* (*n* = 9). To the left of the vertical dashed line are the TP values calculated using Equation 1 (Chikaraishi et al., 2009) and to the right of this line are the TP values calculated using Equation 2 (Nielsen et al., 2015) with multitrophic amino acids (Tr‐AAs). The horizontal dashed line represents the theoretical TP = 2 for primary consumers (Chikaraishi et al., 2009). Asterisks indicate grade of significance in the statistical test (***p < .001; **p < .01)

The TP values were significantly higher for *M. affinis* than for *P. femorata* (*t* test, Ala‐Phe: *t*_1, 15_ = 3.648, *p* < .003; Glu‐Phe: *t*_1,15_ = 2.316, *p* < .04; Figure 1), with the exception of TP calculated from Val‐Phe (*t*_1,15_ = −1.012, *p* > .3). The TP calculated according to Nielsen et al., 2015 (Equation 2) was also significantly higher for *M. affinis* than for *P. femorata* (*t*_1,15_ = 4.204, *p* < .001). Thr‐δ^15^N values differed significantly between the species (*M. affinis*: 1.34 ± 6.10 and *P. femorata*: 8.99 ± 1.06; *t* test, *t*_1,8_ = −2.471, *p* < .05; Figure S6).

**Figure 2:**
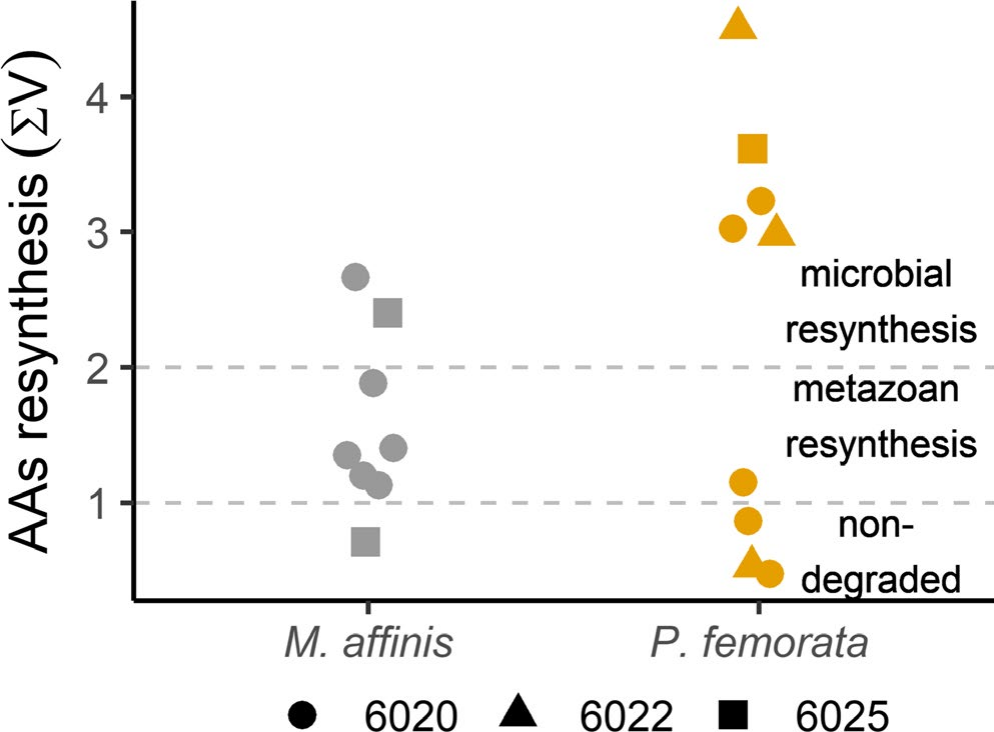
Resynthesis index (∑V) for *Monoporeia affinis* and *Pontoporeia femorata* at the sampling stations. Dotted lines represent the resynthesis index range according to McCarthy et al. (2007). The *P. femorata* subgroups differed significantly from each other (see text)

**Figure 3:**
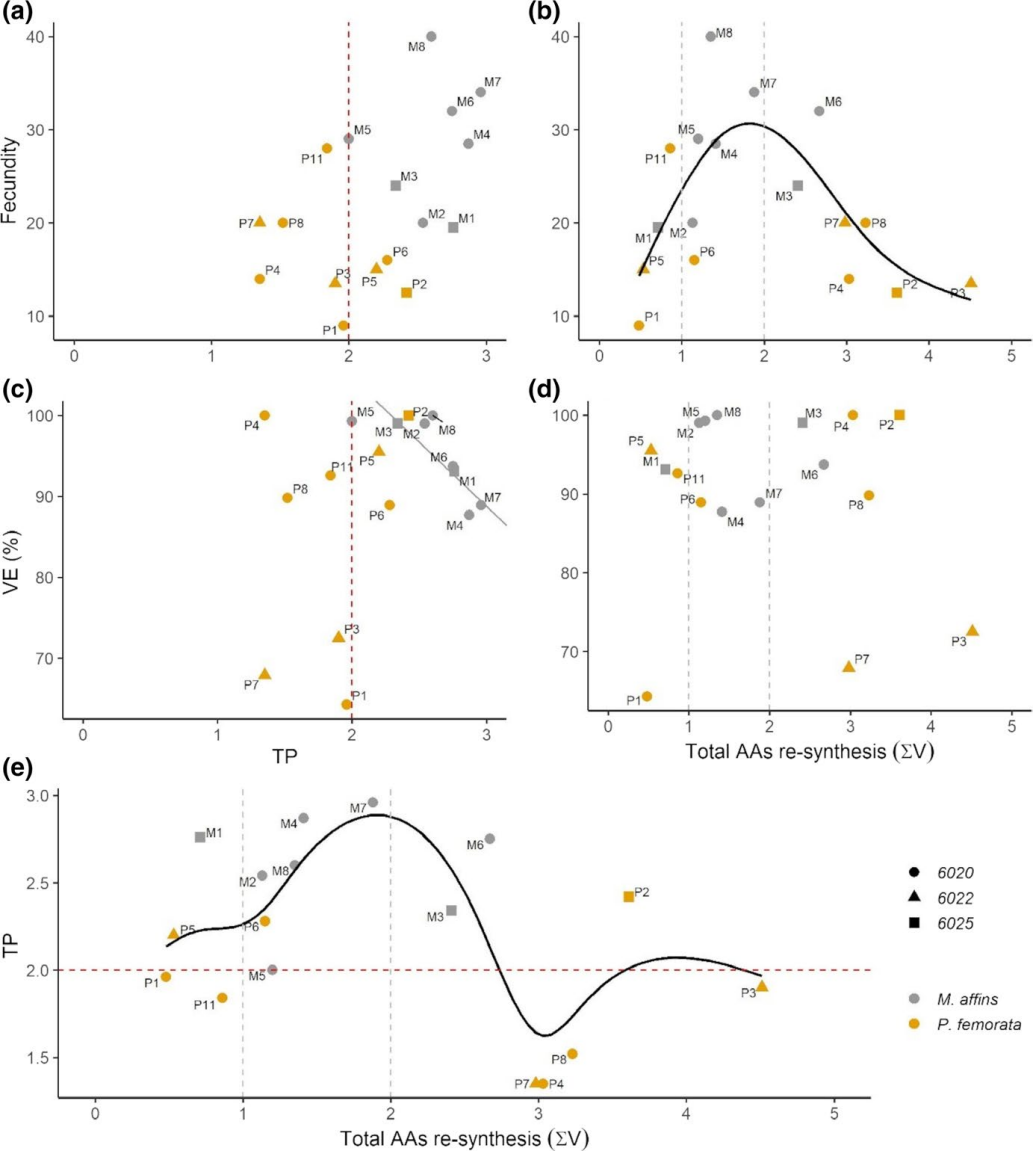
Relationships between the reproductive variables, trophic position (TP) and resynthesis index (∑V) for *Monoporeia affinis*, *Pontoporeia femorata*, and for the amphipods community. Panel a) and c) show fecundity and embryo viability (%VE) in relation to the TP values. In panel b) Fecundity, d) %VE and e) TP are shown in relation to the resynthesis index (∑V). Dashed lines in the panels a, c, and f correspond to the theoretical TP = 2 for amphipods as primary consumers (Chikaraishi et al., 2009). Dashed lines in the panels b, d, and e correspond to the defined range of ∑V in phytoplankton, metazoan, and microbial resynthesis according to McCarthy et al. (2007). Solid lines in gray denote significant relationships and lines in black denote significant at the 0.05 level (*p* > .001); the relationships should, however, be interpreted with caution when degree of freedom are estimated (e.g., using cross‐validation) (Wood 2006). Confidence interval for GAMs models are shown in Figure S8. All graphs show raw data, whereas transformed variables were used in the statistical analyses

The ∑V values varied between and within the species. A significant deviation from unimodality was found for ∑V values in *P. femorata* (HDS = 0.162, *p* < .02; Figure 2, Figure S7). No significant difference in the Phe‐δ^15^N values between the *P. femorata* groups with high and low ∑V values was found (*t*_1,7_ = 0.698, *p* > .5). Neither TP nor % VE nor fecundity differed significantly between the two *P. femorata* groups (Table S4). However, the lack of difference in the TP values between the groups was due to the high value in one sample (P2, Figure 3e) composed by only two individuals with an unusually large biomass; removing this samples resulted in significantly higher TP (*p* < .02) for the group with ∑V < 1.

### 3.2 Relationship between trophic metrics and reproductive status

TP was significantly negatively related to %VE in *M. affinis*, whereas for neither of the species the TP–fecundity relationship was significant (Table 1, Figure 3). For the pooled data (amphipods community), the %VE–TP regression was not significant, whereas TP–fecundity regression was marginally significant (Table 1). In the GAM models, significant smooth terms for fecundity and TP against ∑V were obtained (Table 2, Figure 3), whereas for %VE it was not significant (*R* ^2^ = 0.027, *p* > .9; Table 2, Figure 3). The Thr‐δ^15^N values were significantly negatively correlated to TP in the amphipods community and significantly positively correlated to the resynthesis index (∑V), while no significant correlation was found for fecundity or %VE (Figure S7).

**Table 1:** Standardized major axis (SMA) regressions testing the bivariate relationships between TP and reproductive endpoints; fecundity and percentage of viable embryos in the brood (%VE), for each species (*Monoporeia affinis* and *Pontoporeia femorata*), and for both species together (amphipods community)

Variables	Species	*r_S_*	*p*‐value	b_SMA_ [95%‐CI]
%VE versus TP	*P. femorata*	0.139	.722	0.087 [0.435, 0.174]
	*M. affinis*	−0.826	.013	−0.052 [−0.030, −0.090]
Fec versus TP	*P. femorata*	−0.335	.378	−1.485 [−0.691, −3.193]
	*M. affinis*	0.120	.778	1.992 [ 4.782, 0.830]
%VE versus TP	amphipods community	0.125	.631	2.017 [ 3.378, 1.204]
Fec versus TP	amphipods community	0.458	.064	1.626 [ 2.639, 1.002]

b_SMA_ is the regression coefficient with the associated 95% confidence interval (95% CI, upper and lower limits) in square brackets, and r_S_ is the Spearman rank correlation coefficient with the associated *p*‐value.

**Table 2:** Results of generalized additive models (GAMs) testing effects of the resynthesis index (∑V) on the reproductive variables (fecundity and %VE) and TP in the amphipod community data. The continuos predictor ∑V is used as a smooth term in each regression

Dependent variable	Deviance explained (%)	Adjusted *R* ^2^	*F*‐test	*p*‐value
Fecundity	66	0.509	6.001	.005
%VE	14.7	0.027	0.001	.97
TP	73.2	0.509	3.972	.020

### 3.3 Species and station effects on the reproductive variables

None of the variables tested had a bimodal distribution, although it was close to significant for fecundity in *P. femorata* (HDS = 0.071, *p* > .08; Figure S9). The species differed in reproductive variables and in body mass (BM). *M. affinis* had significantly higher fecundity (U = 66.000, *p* < .005) and %VE (χ^2^_1_ = 6.239, *p* < .02) but lower BM (U = 1.00, *p* < .001) than *P. femorata*. The median values for these traits in *M. affinis* versus. *P. femorata* were as follows: fecundity (29 vs. 15 embryos), %VE (96.4 vs. 89.8% viable embryos in the brood), and BM (1.4 vs. 2.4 mg/ind).

The relationship between fecundity and BM was positive in *M. affinis* (*r_S_* = 0.718, *p* < .001), but not significant for *P. femorata* (*r_S_* = −0.080, *p* > .6; Figure S9). For neither of the species, the correlation between %VE and BM was significant (*M. affinis*: *r_S_* = 0.139, *p* > .4, and *P. femorata:* *r_S_* = −0.214, *p* > .1; Figure S9). Also, the species‐specific correlations between %VE and fecundity were not significant (*M. affinis*: *r_S_* = 0.120, *p* > .4; *P. femorata*: *r_S_* = 0.240, *p* > .1; Figure S9). However, when the station effect was accounted for, significant negative relationships were found for *P. femorata* at stn 6022 and 6025 for %VE‐BM and %VE‐fecundity relationships, respectively, and a positive relationship for *M. affinis* at stn 6020 for the fecundity‐BM relationship (Table S5, Figure S9).

## 4 DISCUSSION

Contrary to our expectations, the two amphipod species rely on the same primary nitrogen source as indicated by the similar δ^15^N‐values in the source amino acid (AA) phenylalanine (Phe). However, both mean values and variation in δ^15^N of the trophic AAs varied significantly between the species suggesting differences in their trophic position. As hypothesized, *Monoporeia affinis* had higher trophic position (TP) than *Pontoporeia femorata*, regardless of the method for TP calculation. In addition, the difference in Thr‐δ^15^N values between the species supports a higher number of trophic transfers in the diet of *M. affinis. P. femorata* showed a greater variability in the resynthesis index (∑V) suggesting a broader feeding niche.

Using isotope labeling approaches, a broader feeding niche has been demonstrated for *P. femorata* in experiments (Byrén et al., 2006; Hill & Elmgren, 1987; Karlson et al., 2010), but our study is the first to show this in the field, since niche analyses using bulk isotope composition was not consistently larger for *P. femorata* in the field during the productive period (Karlson et al., 2014; Karlson, Gorokhova, & Elmgren, 2015). Food availability differs however between seasons at these depths where temperature is always low (around 4–5°C all year below the thermocline; Elmgren, 1978; Siegel, 2008). During winter, when this study was carried out, it is likely that metazoans are the most nutritious food available.

The well‐known difference in the sediment depth distribution between the species (Byrén et al., 2006; Hill & Elmgren, 1987; Karlson et al., 2010) can provide causal explanations to the observed difference in the trophic AA‐metrics. Temporary meiofauna (bivalve spat of *Limecola baltica* and zooplankton resting eggs) have been found in the guts of *M. affinis* (Elmgren, Ankar, Marteleur, & Ejdung, 1986; Sundelin & Elmgren, 1991), and assimilation of carbon derived from zooplankton eggs has been demonstrated (Karlson & Viitasalo‐Frösen, 2009). Although *P. femorata* has not been used in similar experiments, the size range of the food particles is similar to that in *M. affinis* (<60 µm; Ankar, 1977); therefore, it is likely that smaller meiofauna could provide a supplementary food to both amphipods. However, as temporary meiofauna as well as small nematodes and harpacticoids are more abundant in surface sediments (Nascimento et al., 2008; Ólafsson & Elmgren, 1991; Ólafsson, Modig, & van de Bund, 1999), it is likely that they contribute more to the diet of *M. affinis*, resulting in the higher TP values as well as in the lower Thr‐δ^15^N value reflecting more trophic transfers (Figure 1; Figure S10). Lower Thr‐δ^15^N values likely reflect more trophic transfers (Bradley et al., 2015; McMahon et al., 2015; Mompeán et al., 2016) additional support to the higher TP of *M. affinis* comes from the resynthesis index. *M. affinis* had most values between 1 and 2 indicating contribution of amino acids resynthesized by metazoans in the diet, while this was never the case for *P. femorata*, (Figure 2).

An alternative or contributing explanation to the higher TP in *M. affinis* could be the differences in organic matter content and/or microbial communities in the surface and deeper sediment layers, which may affect δ^15^N in sediment AAs (Harris, 1993; Macko & Estep, 1984) and in microorganisms (Calleja, Batista, Peacock, Kudela, & McCarthy, 2013; Fogel, 2019; Fogel & Tuross, 1999; Goedkoop, Åkerblom, & Demandt, 2006). Degrading organic matter can become both enriched and depleted in ^15^N (Calleja et al., 2013; Goedkoop et al., 2006) (Fogel, 2019; Fogel & Tuross, 1999), influencing consumer (including microorganisms) δ^15^N and thus derived trophic metrics. Steffan et al. (2017) compared TP estimates for metazoans (fish and insects) feeding on detritus with high and low microbial (bacteria and fungi) colonization. They found that organisms feeding on detritus enriched with microbes had higher Glu‐δ^15^N values than those feeding on detritus with low levels of microbes. These higher values were directly attributed to the assimilation of microbial AAs by the consumers, which lead to higher TP values. Moreover, it has been found that Ala‐δ^15^N can be used as a tracer of protozoans in diet of mesozooplankton, since only Ala, but not Glu, was elevated in δ^15^N as a result of trophic upgrade by a protozoans (Décima et al., 2017; Gutiérrez‐Rodríguez, Décima, Popp, & Landry, 2014). In our study, the difference between Ala‐δ^15^N and Glu‐δ^15^N was similar between the amphipod species (Table S6, Figure S11); therefore, differential ingestion of protozoans was not likely the main reason for the observed difference in the TP values. Finally, since bacteria are more abundant in surface sediments than deeper down (Llobet‐Brossa et al., 1998) *M. affinis* could be expected to feed more on bacteria than *P.femorata*. However, based on the resynthesis index *M. affinis* did not have a large contribution of bacterially resynthesized AAs in the diet Goedkoop and Johnson (1994) also found bacteria to constitute a negligible part to diet in *M. affinis*. Therefore, the more likely explanation is that metazoans and not microbes contribute to the higher TP in *M. affinis*.

The relative degradation/resynthesis of organic matter, as indicated by the ∑V values, had a similar range (0.3–4.3) to what was found for the deposit‐feeders, including *P. femorata*, from the Chukchi Sea in Canada (Kędra et al., 2019). The same study found that in generally ∑V index increased with TP, although there were several exceptions. Our *P. femorata*, which had generally low TP values than *M. affinis*, had a major contribution of AAs resynthesized by either bacteria or nondegraded organic matter (∑V below 1 and above 3, compared to ∑V of about 2 in Kędra et al., 2019), although this bimodality should be interpreted with caution due to the low sample size. Two possible explanation can be considered, none of them exclusive of another. First, explanation is the compensation with *M. affinis* that often reach high densities and force *P. femorata* to feed deeper down in the sediment (Byrén et al., 2006; Karlson et al., 2010). In this sense, relatively higher TP values observed for *P. femorata* with ∑V below 1 (Figure 3e) could thus be explained by feeding on freshly buried phytodetritus and meiofauna through bioturbation by, for example, *M. affinis* to deeper hypoxic sediment layers where mineralization rate is slower (Bianchi, Johansson, & Elmgren, 2000; Josefson, Forbes, & Rosenberg, 2002; van de Bund et al., 2001). Another explanation is the existence of two subpopulations of *P. femorata* that have different ecological adaptations and occupy different microhabitat, as found for Baltic mysids (Ogonowski, Duberg, Hansson, & Gorokhova, 2013).

Regardless of explanation, *P. femorata* with high ∑V have unrealistically low TP (Figure 3e, three samples were below TP of 1.5). Differences in microbial communities of the sediment and the amphipod gut (Harris, 1993; Larsen et al., 2016) with subsequent effects on isotope fractionation (e.g., the trophic enrichment factor, Δ^15^N) in both microbes (Steffan et al., 2017) and in the amphipods may contribute to the variability in both TP and ∑V between and within species. Low TP values for *P. femorata* with high resynthesis values suggest that the TEF values used (Δ^15^N of 7.6 and 6.6 in Equations 1 and 2, respectively) likely differ between these two diets. The general uncertainty regarding TEF in the AA method has indeed been discussed in several papers (Chikaraishi et al., 2014; McMahon & McCarthy, 2016; Nielsen et al., 2015; Ohkouchi et al., 2017). Consumer‐sediment difference in bulk δ^15^N (a proxy for Δ^15^N in deposit‐feeders) vary among species and is for both amphipods studied here higher in sediment with lower *N*% (Karlson et al., 2015). In the reviews by McMahon and McCarthy (2016) and Ohkouchi et al. (2017), the Δ^15^N Glu‐Phe was found to vary between 0‰ and 10‰ and the variation was attributed to differences in food quality as well as species‐specific mode of nitrogen excretion. Since low metabolism (Cederwall, 1979), low food quality in deeper sediments and bacterial degradation of organic matter ingested (Steffan et al., 2017) would all contribute to produce higher δ^15^N values in trophic amino acids in *P. femorata*, it is likely that the standard Δ^15^N‐values used here results in ecologically erroneous interpretation of TP for species with broad feeding niches.

The observed differences in trophic metrics between species can, with support from the existing experimental studies, be interpreted as resource partitioning with possible implications for both intra‐ and interspecific differences in fecundity, embryo viability, and individual biomass. The two amphipods differ in their life‐history strategies as indicated by the significant differences in the allometric relationship between fecundity and body mass that was positive for *M affinis* but not for *P. femorata*. Contrary to our expectations, there was a negative correlation between embryo viability and TP, although only for *M. affinis*. This species is in contrast to *P. femorata* known to depend on fresh phytodetritus for rapid growth (Karlson, Näslund, Rydén, & Elmgren, 2011). One can speculate that a high contribution of animal prey might cause a stoichiometric mismatch and/or micronutrient deficiency (e.g., vitamins), which could negatively affect embryo development (Pond, Harris, Head, Harbour, 1996).

The generally low embryo viability for *P. femorata*, regardless of the diet could, perhaps, be explained by generally higher stress levels in this species. As a marine amphipod living at the edge of its salinity tolerance in the Baltic Sea, it can experience a chronic osmotic stress with a physiological penalty. In Baltic blue mussels, osmotic regulation is a nitrogen demanding process with costs for growth (Tedengren & Kautsky, 1986). The lack of the allometric‐fecundity relationship for *P. femorata* (in contrast to *M. affinis* which had a positive relationship, like many other invertebrates (Johnson, Stevens, & Watling, 2001; Ramirez Llodra, 2002) could suggest a trade‐off between energy allocated for growth and reproduction. More of the consumed energy might thus be used for osmoregulation and less for fecundity, the former with implications for Δ^15^N and hence resulting δ^15^N values. In arctic waters, *P. femorata* have much higher fecundity than in Baltic Sea (Cederwall & Jermakovs, 1999; Steele & Steele, 1979; Wildsh & Peer, 1981), and a positive relationship between length and fecundity have been observed (Steele & Steele, 1979; Wildsh & Peer, 1981), suggesting that this species is indeed stressed in the Baltic Sea.

In conclusion, we found that the sympatric amphipods which occupy different depths in the sediment have significantly different trophic position (TP) and also differ in the resynthesis index (∑V), as estimated by nitrogen isotope composition of amino acids. The surface‐feeding amphipod *Monoporeia affinis* had higher TP, which may indicate a higher contribution of animal prey in the diet compared to the sub‐surface‐feeding *Pontoporeia femorata*, which appears to have a diet dominated by either nondegraded or bacterially degraded organic matter. More studies are, however, needed to determine whether a higher degree of carnivorous feeding for *M. affinis* is supporting successful reproduction.

## CONFLICT OF INTEREST

None declared.

## AUTHOR CONTRIBUTION

**Matias Ledesma:** Conceptualization (equal); Data curation (lead); Formal analysis (lead); Investigation (equal); Methodology (equal); Project administration (equal); Validation (equal); Visualization (lead); Writing‐original draft (lead). **Elena Gorokhova:** Conceptualization (equal); Formal analysis (supporting); Funding acquisition (equal); Investigation (equal); Methodology (equal); Project administration (equal); Resources (equal); Visualization (supporting); Writing‐original draft (supporting); Writing‐review & editing (equal). **Henry Holmstrand:** Resources (equal); Software (supporting); Writing‐review & editing (supporting). **Andrius Garbaras:** Resources (equal); Software (supporting); Writing‐review & editing (supporting). **Agnes ML Karlson:** Conceptualization (equal); Formal analysis (supporting); Funding acquisition (equal); Investigation (equal); Methodology (equal); Project administration (equal); Resources (equal); Validation (lead); Visualization (supporting); Writing‐original draft (supporting); Writing‐review & editing (equal).

## Supporting information

AppendixS1

## Data Availability

Datasets supporting this article are submitted to the Dryad Digital Repository and will be available upon acceptance of the manuscript (https://doi.org/10.5061/dryad.9zw3r22b3).
