# Supplementary material for: Nitrogen isotope composition of amino acids reveals trophic partitioning in two sympatric amphipods
Source: Ecol Evol. 2020 Sep 23;10(19):10773–84. doi: 10.1002/ece3.6734 (PMC7548185; doi:10.1002/ece3.6734)
Supplement: Supplementary file 1 — AppendixS1 [file ECE3-10-10773-s001.docx]

**Appendix:**

**Nitrogen isotope composition of amino acids reveals trophic partitioning in two sympatric amphipods**

**Content (in the order they appear in manuscript):**

**1) Figures S1-S11**

**2) Tables S1 – S6**

**3) Supplementary method description (page 5)**


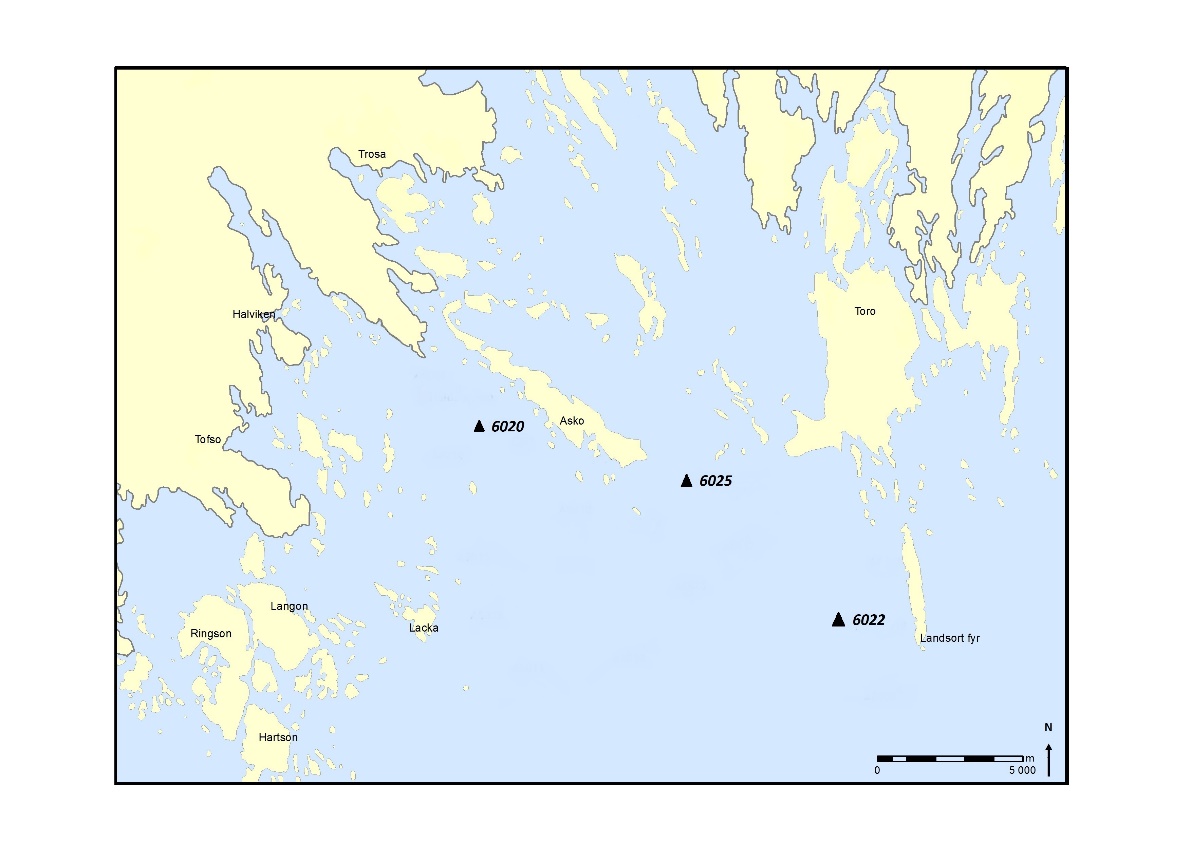


**Figure S1**: Detailed map of the study area in the southern Stockholm Archipelago, Sweden (approx. 80 km south of Stockholm) with the stations included in the study and the island Askö, where the field station of Stockholm University is located (58° 49.5' N, 17° 39' E). These stations are also included in the National Monitoring Program for monitoring biological effects of contaminated sediments.

**
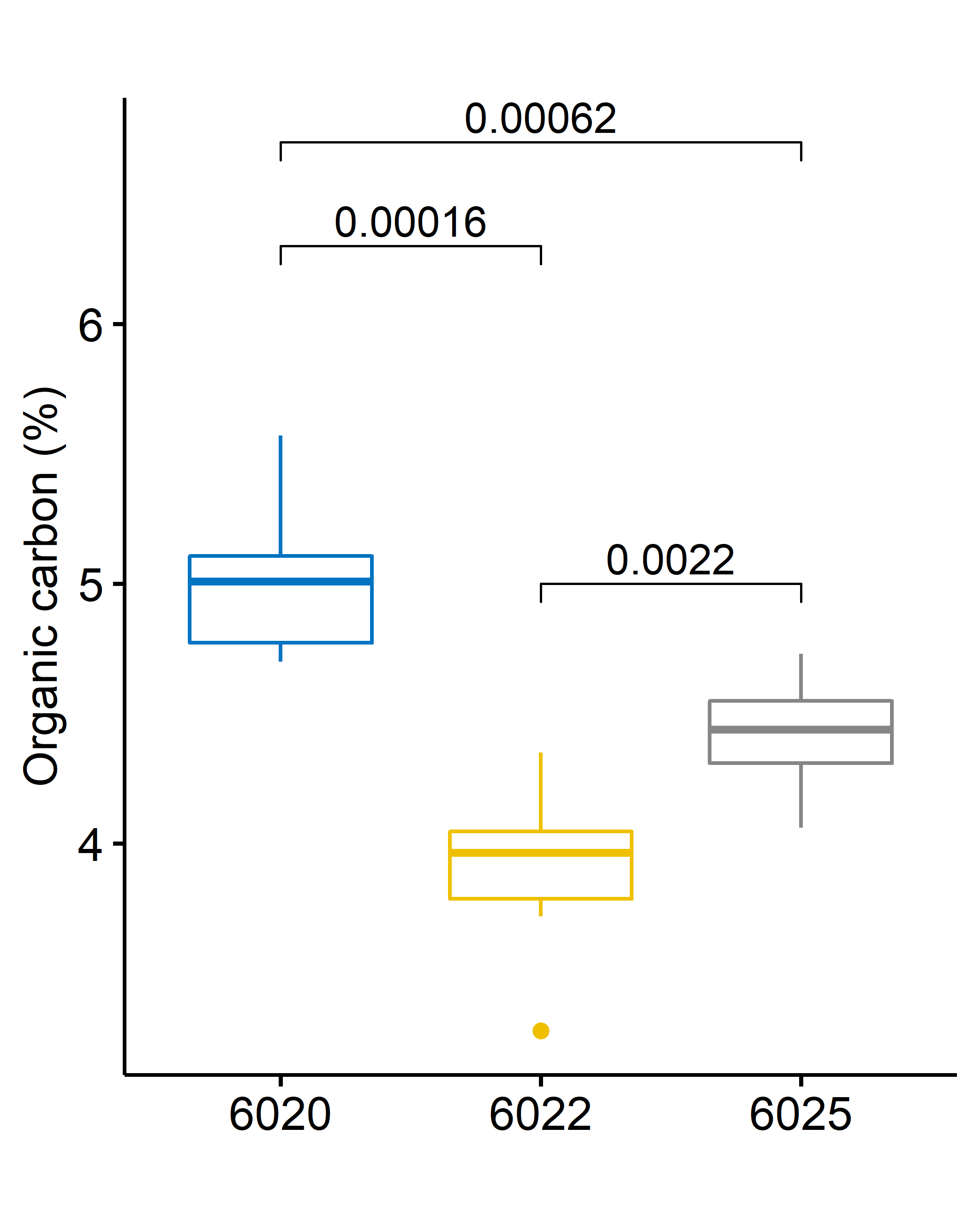
**

**Figure S2**: Total organic carbon (% of dry mass) in surface sediment (0-2 cm) collected between 2005-2012 during the National Monitoring Program for monitoring biological effects of contaminated sediments. Outlier value for station 6022 corresponds to year 2012. Horizontal square brackets between stations show p-values according to Tukey post-hoc tests.


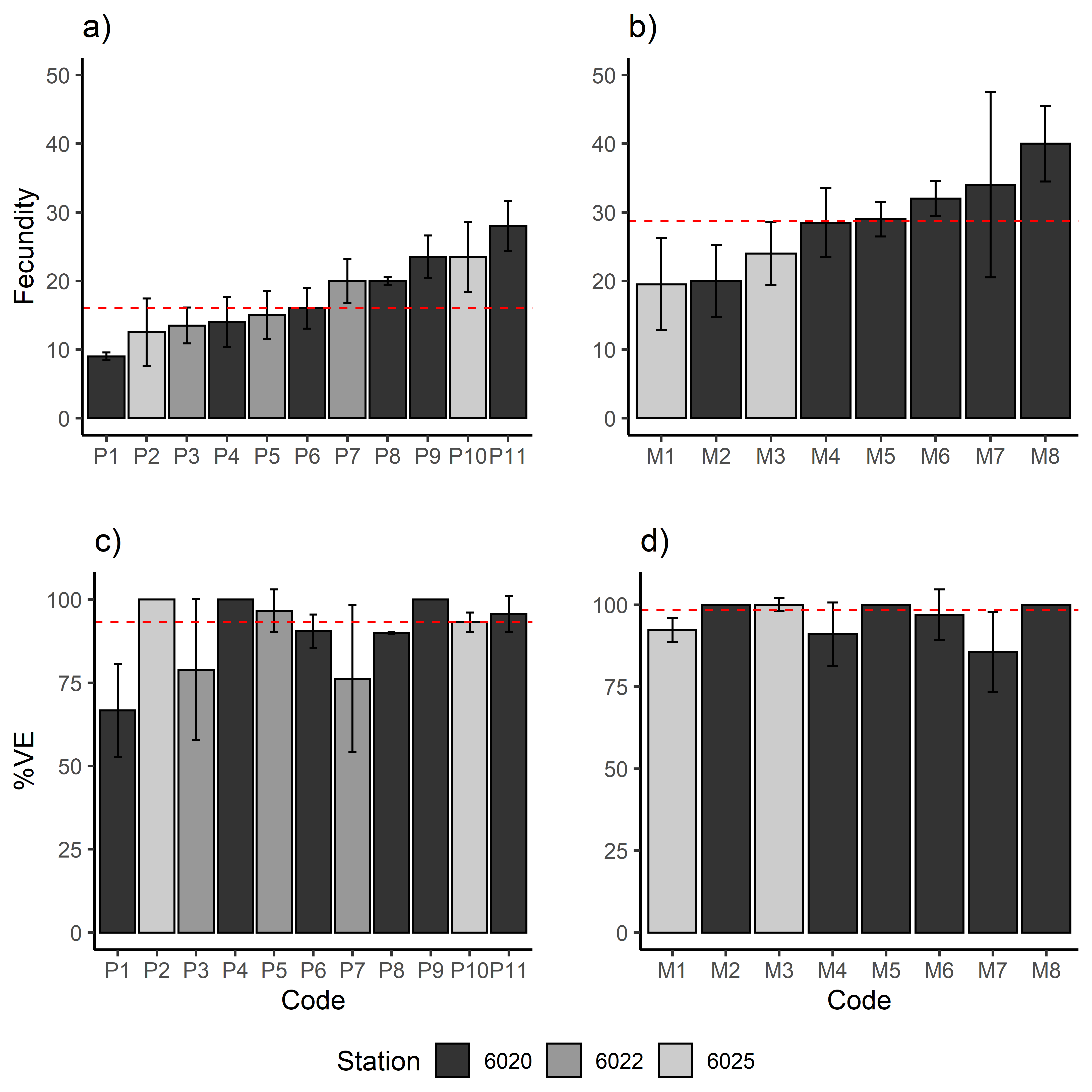


**Figure S3**: Fecundity and percentage viable embryos (%VE) for the two amphipod species and stations in samples used for SIA. Codes P1-P11 represent *Pontoporeia femorata* samples (panel a, c) and M1-M8 *Monoporeia affinis* samples (panel b, d). Samples are ordered by fecundity in ascending order. Values for fecundity are mean and standard error and median and confidence interval for %VE. Red dashed line corresponds to the median fecundity and %VE across the dataset.

**Quality control and applied corrections of chromatograms**

The chromatograms were visually inspected before extracting the isotopes values. When the peaks were not clearly separated due to co-elution, each peak was redefined manually and, if the isotope ratio of the new peak remained within the standard deviation, it was retained. The intensity range 100-200 mV was considered optimal. However, higher intensity up to 1000 and down to 50 mV were also used when necessary, which still gives acceptable precision. The linearity was good, meaning that there was no significant correlation between intensity and δ^15^N in the range measure before (50 – 1000 mV).


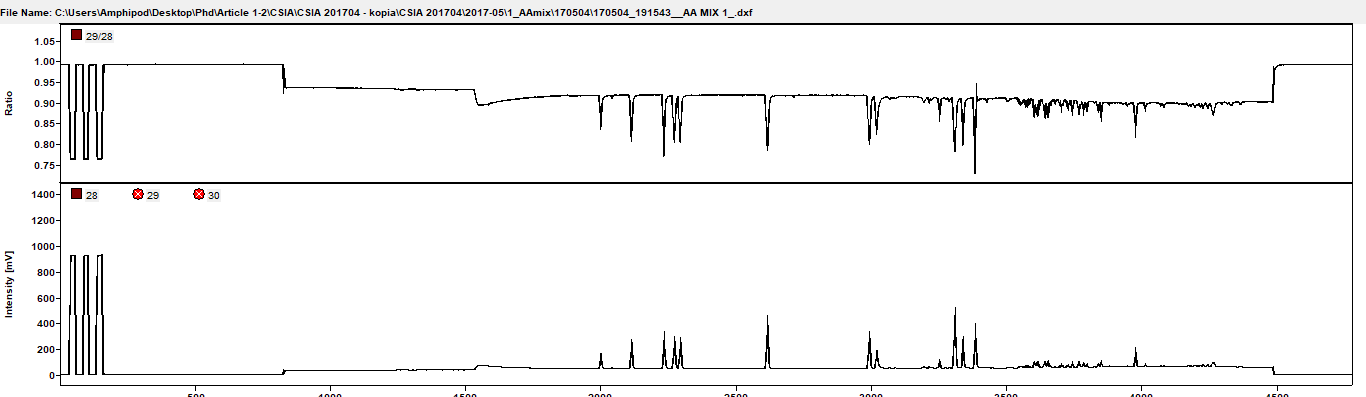


Time [s]

**4**

**3**

**2**

**1**

**Figure S4**. GC – C – IRMS chromatogram of the internal standard. Components: valine (1), alanine (2), phenylalanine (3) and pyro-glutamic acid (4).

The analytical standard deviation (mean = 1.26 ‰, SD =0.49) was calculated using equal-volume single injections of the same sample; this gives a better estimate of the reproducibility than multiple injections from the same sample (H. Holmstrand pers. comm). Correction for retention time and drift was based on bracketed external standards of amino acids (AAs) with known isotopic composition. Correction due to time was applied in those cases where the difference in δ^15^N values between standards was higher than 2 ‰_._ Otherwise, only correction for the drift was applied.

First, we evaluated whether the addition of the external standard norleucine (Nle) to the standard samples may have altered the isotopic values of the AAs, and consequently the calculation of the trophic position (TP). Brackets (Table S1) were structured with double standards in each end, arranged with presence / absence of Nle so it was possible to compare both pairs of standard combinations (with and without Nle) to correct the same sample, using t-test. The same approach was applied to test potential effects on Nle on the calculated TP values. We found no difference between the isotopic composition ​​and the derived TP from the same sample (Table S1), corrected with and without Nle, meaning that it was possible to use all samples regardless if they were corrected from brackets with or without Nle.

**Table S1**. Differences in δ^15^N of trophic AAs and derived TP between samples corrected for time (drift), with and without presence of norleucine (Nle) (see above). AAs: alanine (Ala); pyro glutamic acid (Glu); valine (Val).

**Table S2**. δ^15^N values of trophic amino acids (AAs) from *Monoporeia affinis* (M3-M6) and *Pontoporeia femorata* (P2-P10) samples used for resynthesis index (∑V) calculation, according to eq. 3 (McCarthy et al., 2007). There is no significant differences in ∑V calculated using 3 AAs (Glutamic acid, Alanine and Valine) and 6 AAs (Paired t- test, t_1, 7_ = -0.225, p > 0.4). Only for sample P2 did the calculation with 6 AAs change the categorisation obtained when calculating it with 3 AAs (from category >2 with 3 AAs to category 1-2 with 6 AAs).

**
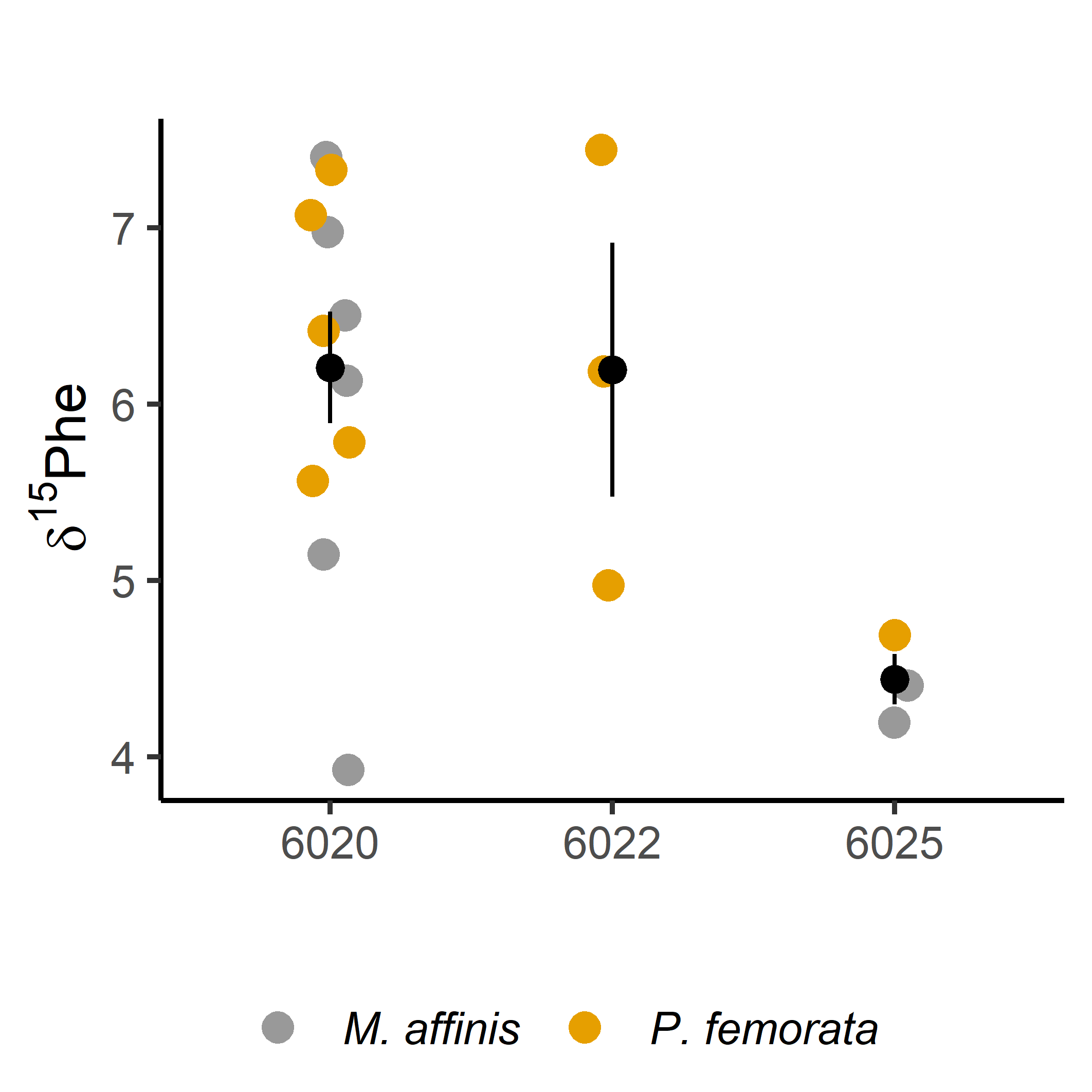
**

**Figure S5***.* δ^15^N values for Phenylalanine (Phe) at the sampling stations for *Monoporeia affinis* and *Pontoporeia femorata*. Black circles and error bars represent grand mean value ± standard error for Phe δ^15^N for all amphipod specimens within a station.

**Table S3.** Results from one-way ANOVA (F–test) and Kruskal-Wallis (χ^2^-test), testing differences in δ^15^N-trophic-AAs (Tr-AAs) for amphipods among stations (6020, 6022 and 6025).

**
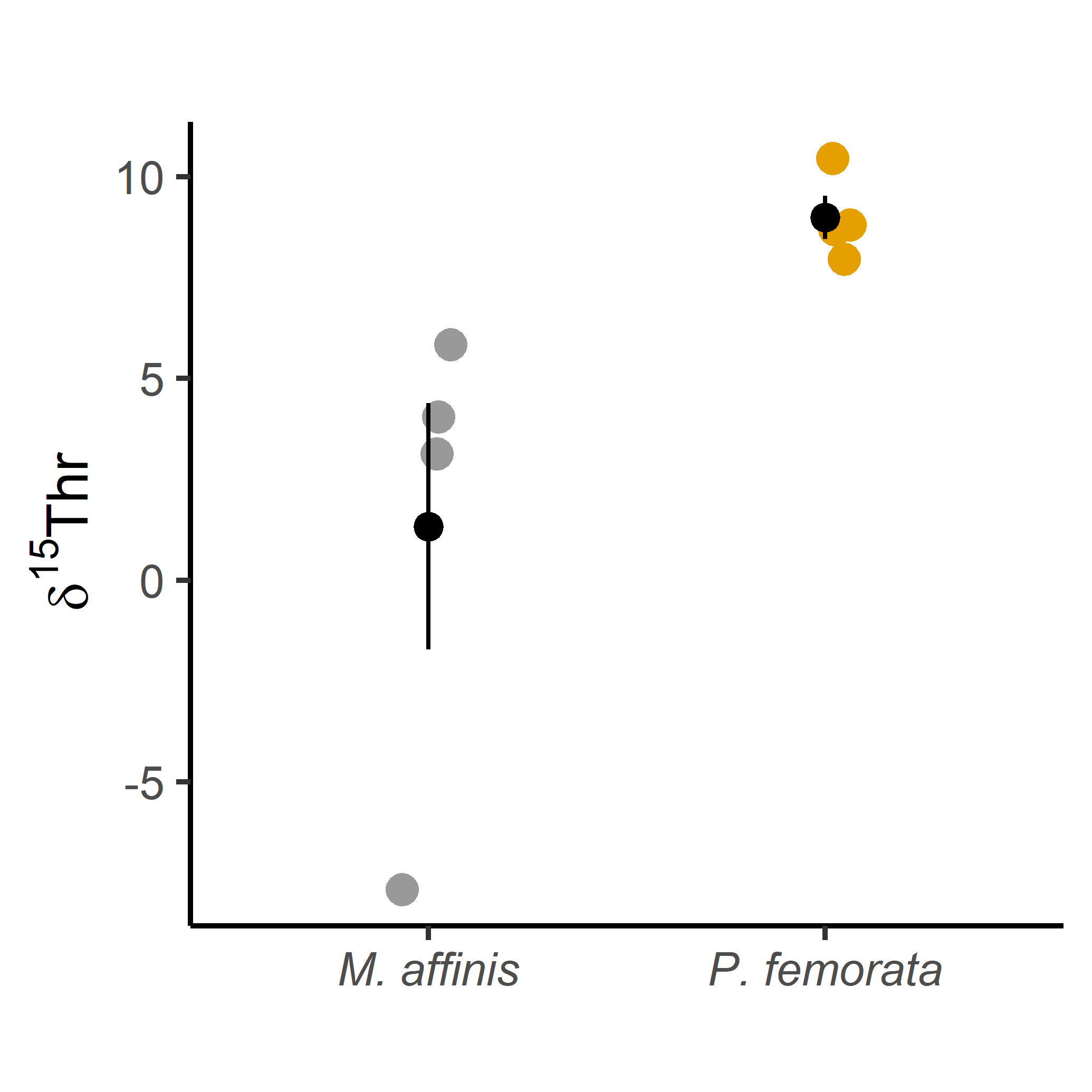
**

**Figure S6:** δ^15^N-Threonine (Thr) for *Monoporeia affinis* and *Pontoporeia femorata*. Black circles and error bars represent grand mean value ± standard error for Thr-δ^15^N for each species.

**
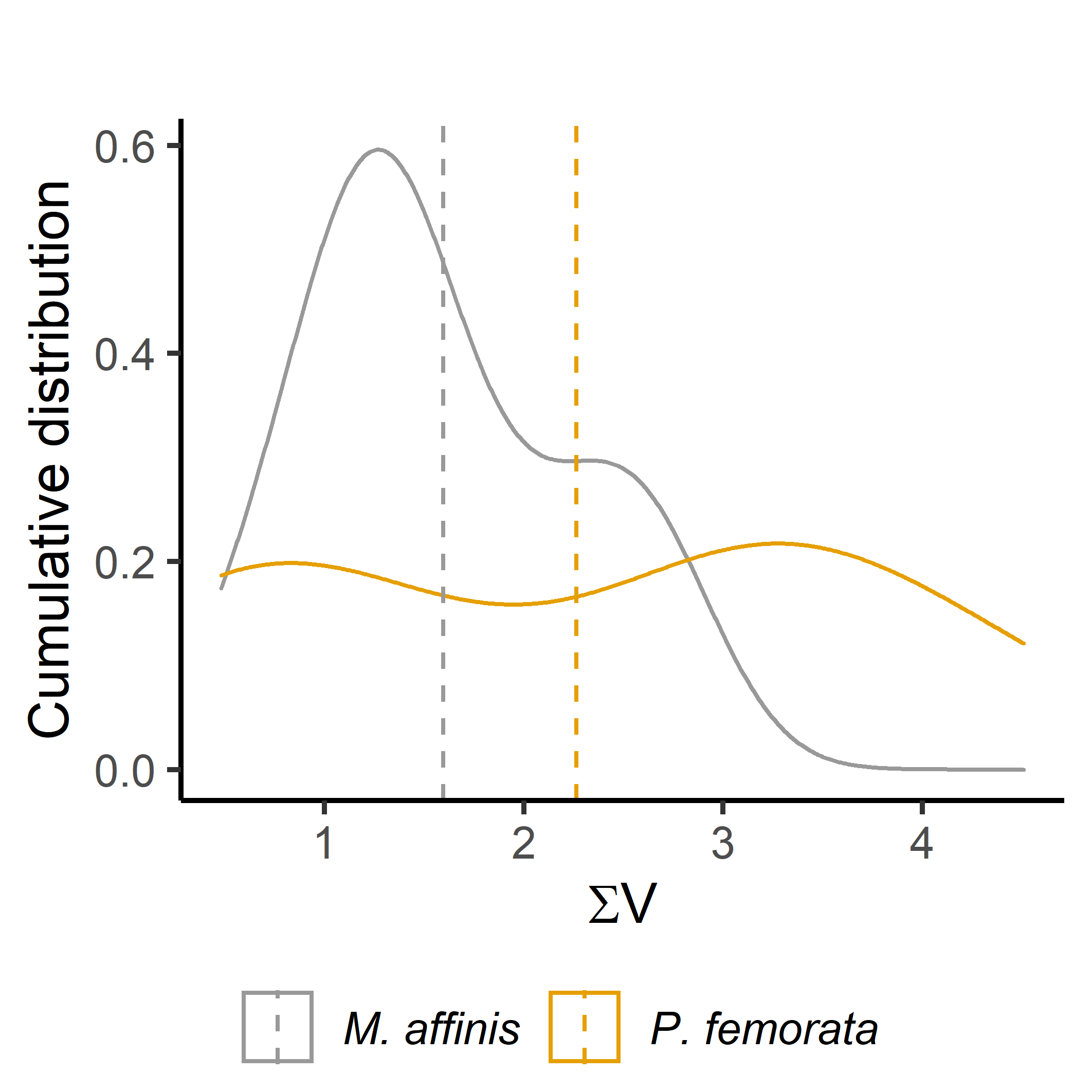
**

**Figure S7.**  Resynthesis index (∑V) density estimation for *Monoporeia affinis* and *Pontoporeia femorata*. The vertical dashed lines represent the mean value for each species.

**Table S4.** Differences in trophic position (TP) and reproductive variables (Fecundity and percentage viable embryos (%VE)) between both sub-populations of *Pontoporeia femorata,* tested with unpaired t-test or Chi-square test (χ^2^).

**
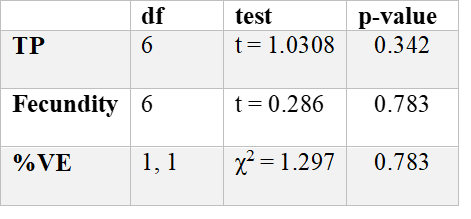
**

**
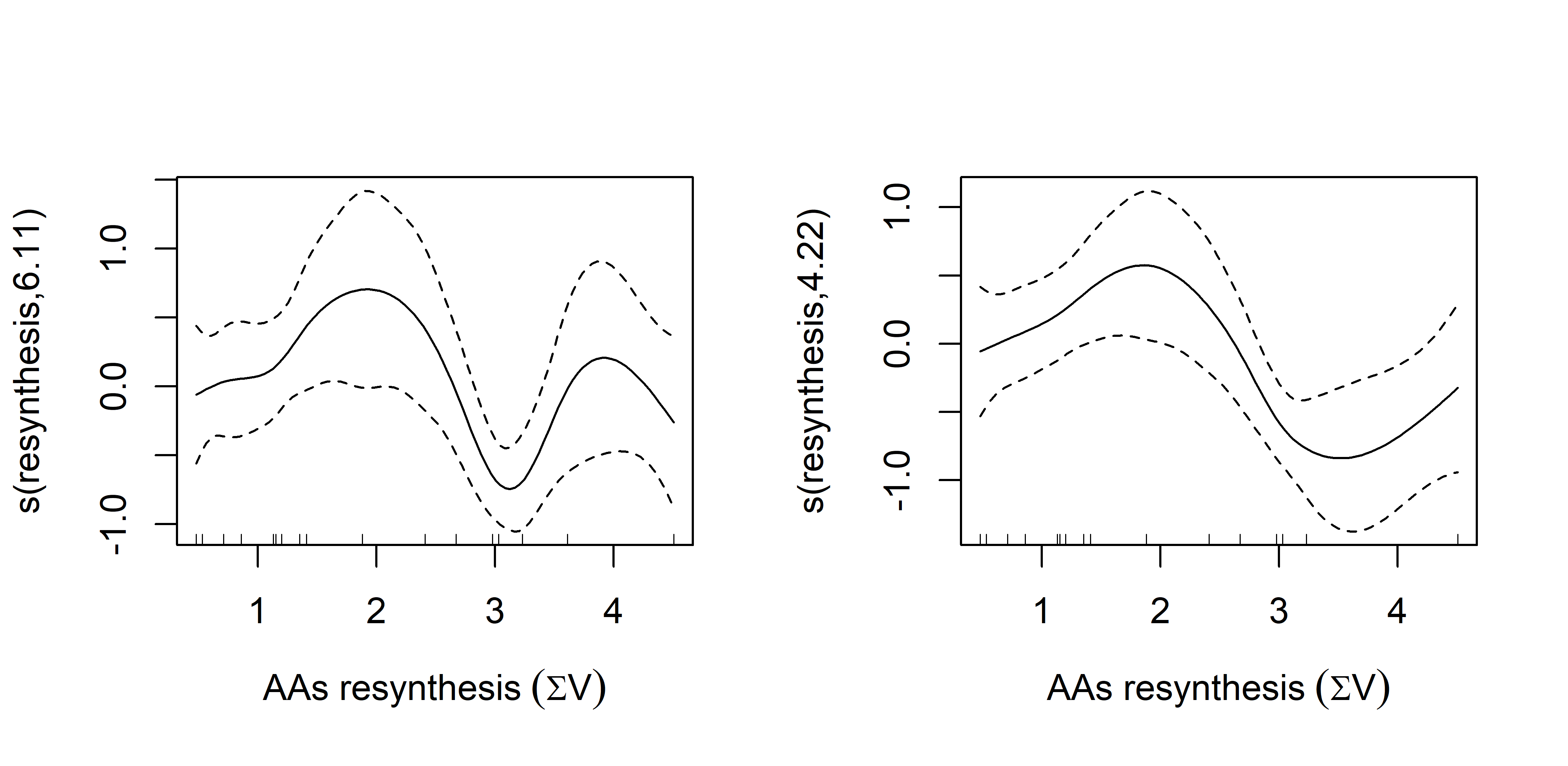
**

**Figure S8.** Estimated smoothing curve. The x-axis shows the values of resynthesis index (∑V) and the y-axis the contribution of the smoother to the fitted values, modelled with a Generalized Linear Model (GAM). The credible intervals are shown (added with a Bayesian approach by the mgcv package (Wood, 2019). The analysis in the left panel include outlier sample P2 while this sample is removed in the right panel; the smooth term (∑V) increases then significantly.

**
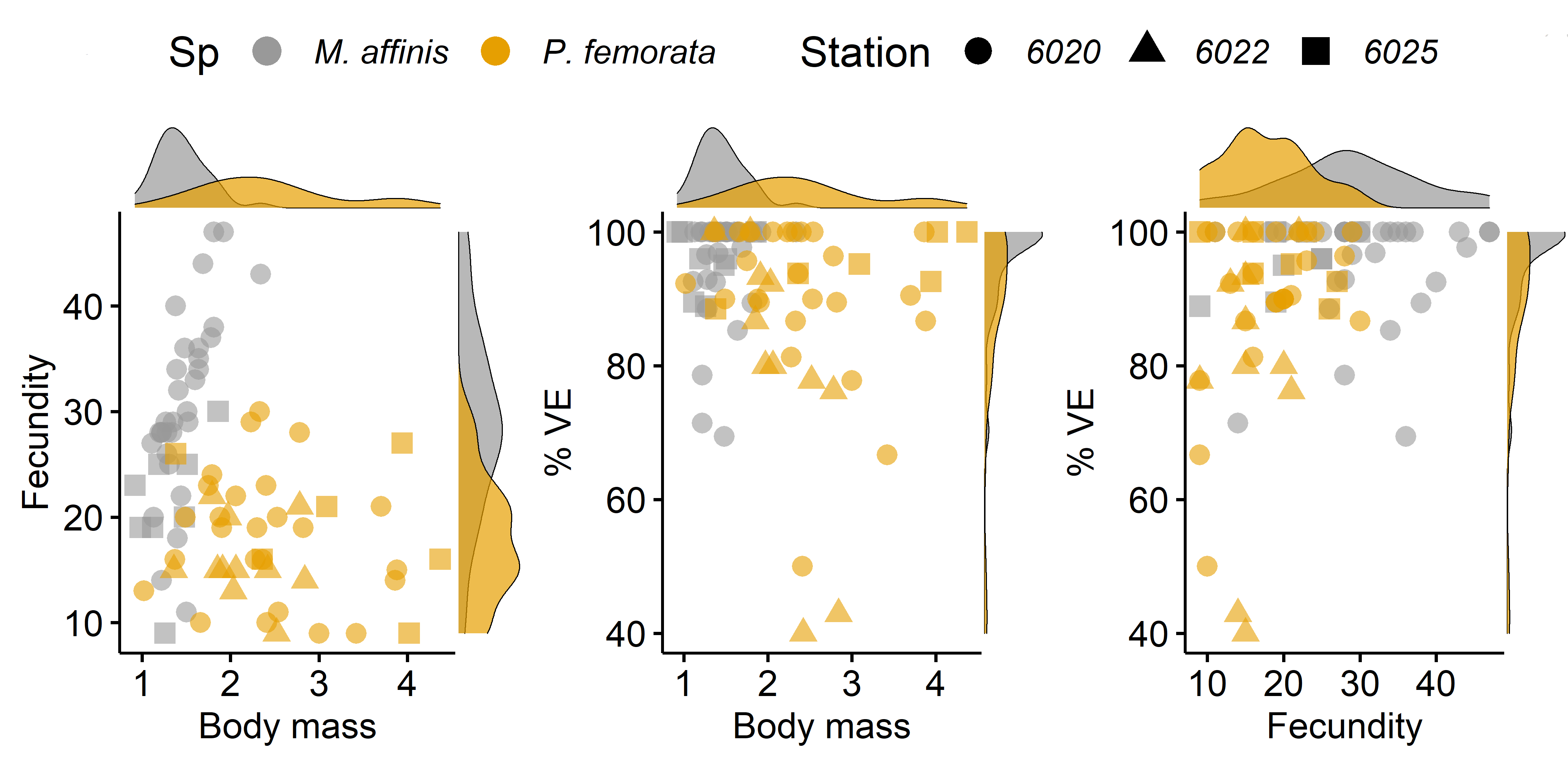
**

**Figure S9**. Distribution of physiological variables used for standardized minor axis regression (Warton et al., 2006) based on the individual data for *Monoporeia affinis* and *Pontoporeia femorata*. From left: Fecundity against body mass, %VE against body mass and %VE against fecundity, for each species and the three stations.


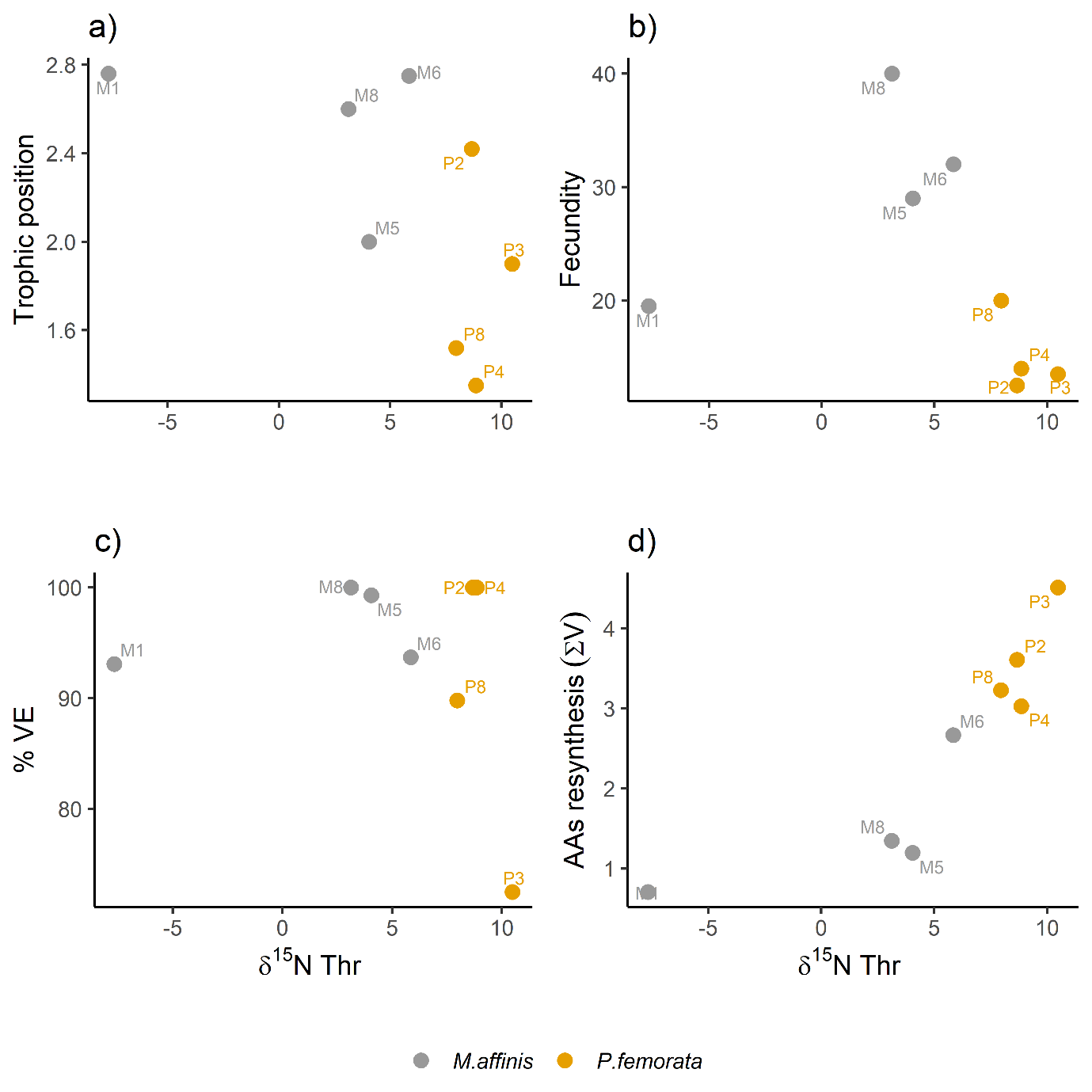


**Figure S10**: Relationship between trophic position (TP), reproductive variables and the resynthesis index (∑V) against Threonine-δ^15^N (Thr-δ^15^N) for *Monoporeia affinis* and *Pontoporeia femorata* as amphipod data. Significant correlations for amphipods were found between TP and Thr-δ^15^N (panel a, r_S_= -0.738, p = 0.045) and between ∑V and Thr-δ^15^N (panel d, r_S_= 0.904, p = 0.004). No significant correlation was found for fecundity (panel b, r_S_= -0.666, p = 0.083) or VE% (panel c, r_S_= -0.121, p= 0.773) against Thr-δ^15^N. Please note that Thr-δ^15^N values for *P. femorata* samples originate only from the subgroup with high ∑V-values.

**Table S5**: Station-specific SMA regression model for the relationship between physiological status variables for individual data: fecundity vs body mass (BM), percentage viable embryos (%VE) vs BM and %VE vs fecundity. Values in square brackets represent 95% confidence interval (95%-CI, upper and lower limits).

**
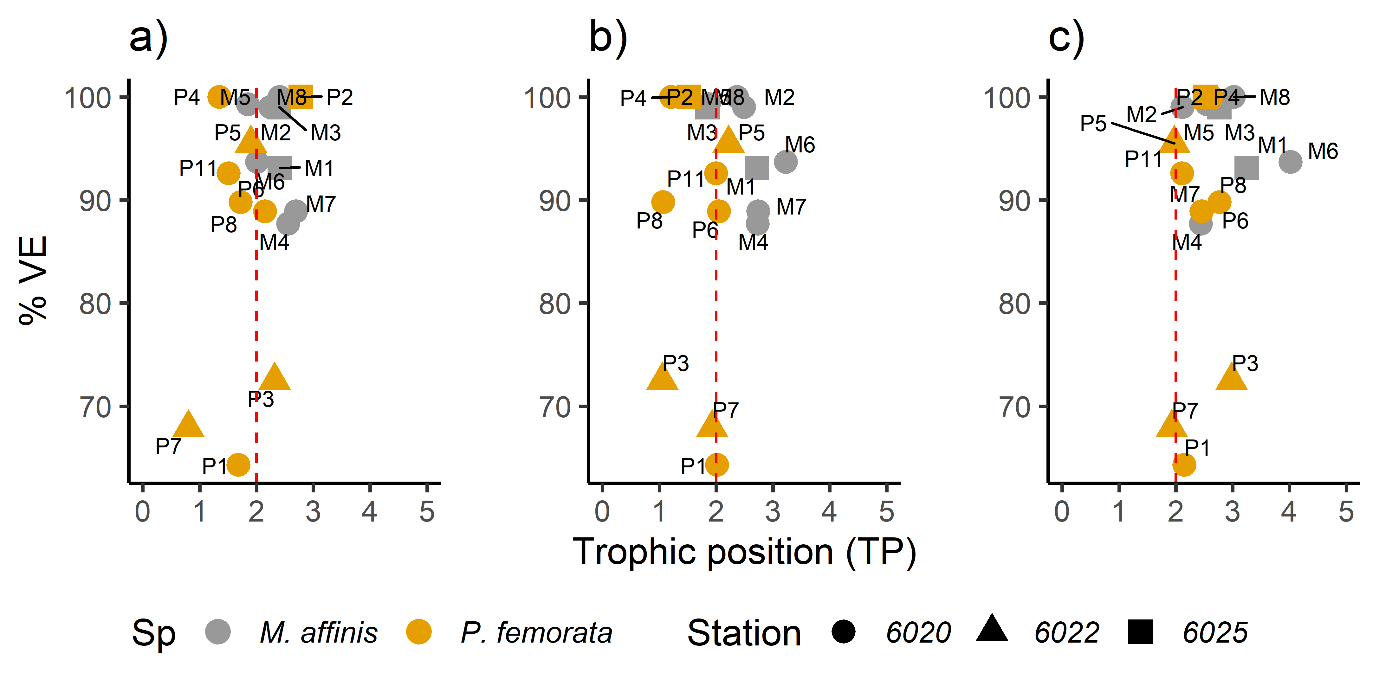
**

**Figure S11.** Percentage of viable embryos (VE %) plotted against trophic position (TP) of the samples for each species and station. TP was calculated from a single pair of AAs (a) Glu – Phe, (b) Ala – Phe and (c) Val – Phe; the calculations were conducted according to Chikaraishi et al. (2009). The vertical dashed red line represents the theoretical TP = 2 for primary consumers.

**Table S6:** Trophic position (TP) calculated for glutamic acid (Glu) and alanine (Ala) using parameters from Chikaraishi et al. (2009) and the differences between them (∆TP)**.**

**References:**

Chikaraishi, Y., Ogawa, N. O., Kashiyama, Y., Takano, Y., Suga, H., Tomitani, A., … Ohkouchi, N. (2009). Determination of aquatic food-web structure based on compound-specific nitrogen isotopic composition of amino acids. Limnology and Oceanography: Methods, 7(11), 740–750. doi: 10.4319/lom.2009.7.740

McCarthy, M. D., Benner, R., Lee, C., & Fogel, M. L. (2007). Amino acid nitrogen isotopic fractionation patterns as indicators of heterotrophy in plankton, particulate, and dissolved organic matter. Geochimica et Cosmochimica Acta, 71(19), 4727–4744. doi: 10.1016/j.gca.2007.06.061

Warton, D. I., Wright, I. J., Falster, D. S., & Westoby, M. (2006). Bivariate line-fitting methods for allometry. Biological Reviews, 81(2), 259–291. doi: 10.1017/S1464793106007007

Wood, S. N. (2019). mgcv: Mixed GAM Computation Vehicle with Automatic Smoothness Estimation. R package version 1.8-31. https://cran.r-project.org/packages=mgcv (2019). Retrieved January 30, 2020, from <https://cran.rproject.org/web/packages/mgcv/citation.html>
